# Supplementary material for: An RBD-Based Diagnostic Method Useful for the Surveillance of Protective Immunity against SARS-CoV-2 in the Population
Source: Diagnostics (Basel). 2022 Jul 5;12(7):1629. doi: 10.3390/diagnostics12071629 (PMC9324632; doi:10.3390/diagnostics12071629)
Supplement: Supplementary file 1 [file diagnostics-12-01629-s001.zip › diagnostics-1683151-supplementary.pdf]

```
Bacterial       -----------mnitnlcpfgevfnatrfasvyawnrkrisncvadysvlynsasfstfk    49
Eukaryotic      rvqptesivrfpnitnlcpfgevfnatrfasvyawnrkrisncvadysvlynsasfstfk    60
                           *************************************************

Bacterial       cygvsptklndlcftnvyadsfvirgdevrqiapgqtgkiadynyklpddftgcviawns    109
Eukaryotic      cygvsptklndlcftnvyadsfvirgdevrqiapgqtgkiadynyklpddftgcviawns    120
                ************************************************************

Bacterial       nnldskvggnynylyrlfrksnlkpferdisteiyqagstpcngvegfncyfplqsygfq    169
Eukaryotic      nnldskvggnynylyrlfrksnlkpferdisteiyqagstpcngvegfncyfplqsygfq    180
                ************************************************************

Bacterial       ptngvgyqpyrvvvlsfellhapatv-----------------     195
Eukaryotic      ptngvgyqpyrvvvlsfellhapatvcgpkkstnlvknkcvnf     223
                **************************
```

Figure S1. Alignment of the RBD amino acid sequences used for bacterial expression in BL21(DE3) cells or eukaryotic expression in mammalian HEK293T cells. Asterisks show identical amino acids.
